# The Proteome of Equine Oviductal Fluid Varies Before and After Ovulation: A Comparative Study

**DOI:** 10.3389/fvets.2021.694247

**Published:** 2021-08-05

**Authors:** Pablo Fernández-Hernández, Federica Marinaro, María Jesús Sánchez-Calabuig, Luis Jesús García-Marín, María Julia Bragado, Lauro González-Fernández, Beatriz Macías-García

**Affiliations:** ^1^Research Group of Intracellular Signaling and Technology of Reproduction (Research Institute INBIO G+C), University of Extremadura, Cáceres, Spain; ^2^Department of Animal Medicine, Faculty of Veterinary Sciences, University of Extremadura, Cáceres, Spain; ^3^Stem Cell Therapy Unit, Jesús Usón Minimally Invasive Surgery Centre, Cáceres, Spain; ^4^Department of Animal Medicine and Surgery, Faculty of Veterinary Sciences, University Complutense of Madrid, Madrid, Spain; ^5^Department of Physiology, Faculty of Veterinary Sciences, University of Extremadura, Cáceres, Spain; ^6^Department of Biochemistry and Molecular Biology and Genetics, Faculty of Veterinary Sciences, University of Extremadura, Cáceres, Spain

**Keywords:** horse, oviductal fluid, ovulation, mass spectrometry, proteome, domestic animal reproduction

## Abstract

Equine fertilization cannot be performed in the laboratory as equine spermatozoa do not cross the oocyte's zona pellucida *in vitro*. Hence, a more profound study of equine oviductal fluid (OF) composition at the pre-ovulatory and post-ovulatory stages could help in understanding what components are required to achieve fertilization in horses. Our work aimed to elucidate the proteomic composition of equine OF at both stages. To do this, OF was obtained postmortem from oviducts of slaughtered mares ipsilateral to a pre-ovulatory follicle (*n* = 4) or a recent ovulation (*n* = 4); the samples were kept at −80°C until analysis. After protein extraction and isobaric tags for relative and absolute quantification (iTRAQ) labeling, the samples were analyzed by nano-liquid chromatography coupled to tandem mass spectrometry (LC-MS/MS). The analysis of the spectra resulted in the identification of a total of 1,173 proteins present in pre-ovulatory and post-ovulatory samples; among these, 691 were unique for *Equus caballus*. Proteins from post-ovulatory oviductal fluid were compared with the proteins from pre-ovulatory oviductal fluid and were categorized as upregulated (positive log fold change) or downregulated (negative log fold change). Fifteen proteins were found to be downregulated in the post-ovulatory fluid and 156 were upregulated in the post-ovulatory OF compared to the pre-ovulatory fluid; among the upregulated proteins, 87 were included in the *metabolism of proteins* pathway. The identified proteins were related to *sperm–oviduct interaction, fertilization*, and *metabolism*, among others. Our data reveal consistent differences in the proteome of equine OF prior to and after ovulation, helping to increase our understanding in the factors that promote fertilization and early embryo development in horses.

## Introduction

Assisted reproductive technologies (ARTs) are commonly used in the field of reproduction to obtain embryos in domestic species (1). Currently, *in vitro* fertilization (IVF) is the technique of choice to produce embryos *in vitro* in domestic species such as bovine (2). The first report of unequivocal successful IVF in domestic species was published in rabbits in 1954 (3, 4). In this report, the authors used spermatozoa recovered from the female's reproductive tract after artificial insemination to perform IVF (3). Until that moment, IVF failure was associated with the lack of adequate induction of sperm capacitation, a maturational process in which the spermatozoon needs to fully acquire its fertilizing capacity (4, 5). Since then, fertilization media were designed for each species based on the composition of the reproductive fluids, which consistently vary among species (bovine, human, mice, and porcine) (6, 7). Unfortunately, despite the efforts of different research groups around the world, the *in vitro* production of equine embryos is still very inefficient, in part due to the low success rate of *in vitro* fertilization that varies from 0 to 33% (8, 9).

Recent research demonstrates that the failure of equine IVF is most likely attributable to the inability of the spermatozoa to penetrate the oocyte's zona pellucida (10) and, hence, to a suboptimal composition of the fertilization media that results in ineffective sperm capacitation (9, 11). This theory is supported by the fact that oviductal transfer of *in vitro* matured equine oocytes in an inseminated mare results in embryonic development at similar rates to those obtained from spontaneous ovulations (12). Interestingly, it has been described that equine IVF conditions support the binding of stallion spermatozoa to the zona pellucida, but they fail to induce the acrosome reaction and other capacitation-related events (8). Therefore, mimicking the conditions of the oviductal environment could be the key for successful equine IVF as it provides the ideal microenvironment for fertilization, promoting adequate sperm capacitation (8). A deeper understanding of the composition of oviductal secretions is required to better mimic the oviductal milieu and to ensure sperm capacitation *in vitro* in the horse.

Oviductal fluid (OF) is composed of ions, hormones, growth factors, metabolites, and proteins, among other compounds (6). The oviduct is lined with an epithelium coated by OF, which is composed of secretions of these cells and of blood plasma filtrate (11). Hence, the oviduct provides a dynamic microenvironment that changes according to the stage of the estrus cycle (13), ovulation site (14), oviductal region (15), and the presence of gametes or embryos (16). At present, the addition of natural reproductive fluids to commercial IVF media has been shown to improve embryo quality and yield in cows (2), highlighting the importance of studying the composition of oviductal secretions to improve current ARTs in domestic species (17).

The composition of the OF greatly varies depending on the species in study; hence, the results obtained from a single species cannot be extrapolated to others (18). In the particular case of horses, the difficult anatomical approach of the oviduct, the limited number of equine slaughterhouses, and the low amount of OF produced (11) render the addition of native oviductal fluid to the IVF media unfeasible in horses.

For these reasons, to fully develop an equine IVF protocol and to increase the embryo yield and the quality of the embryos produced *in vitro*, a more profound study of the physiology of the oviduct and the composition of its secretions is of outmost importance (8). Previous studies have reported the metabolome of equine OF (11) or the proteome of OF in mares during early embryo development (19), but more research is required to fully unravel the key factors leading to successful fertilization *in vitro* in horses.

One of the main components of the OF are proteins, but their functions are still under study (6, 18). In horses, consistent differences in the proteomic composition of the oviductal secretions in pregnant mares compared to non-pregnant counterparts, and also in the ipsilateral and contralateral oviduct where the embryo is allocated, have been described (19). In this regard, some authors claim that unsuccessful *in vitro* fertilization is related to the lack of specific proteins in the fertilization medium that may be impeding complete sperm capacitation (6).

Thus, in the present work, we aimed to elucidate the proteomic composition of equine OF at the pre-ovulatory and post-ovulatory stages. Besides, the different protein compositions between both stages were also contrasted. Our results show that the proteome of equine OF varies prior to and post-ovulation. Interestingly, enrichment analysis of the upregulated proteins revealed that 15 proteins were found to be downregulated and 156 were upregulated in post-ovulatory OF compared to pre-ovulatory OF. Using the enrichment analysis approach, the main categories identified were directly related with metabolism. The Reactome pathway analysis showed that, in the proteins identified as upregulated in the post-ovulatory OF, 87 of them were included in the *metabolism of proteins* pathway and 56 were enclosed in the *developmental biology* pathway, revealing an intense protein turnover during early embryo development in horses.

## Materials and Methods

### Collection of Oviductal Fluid

Oviducts were obtained immediately postmortem at a commercial slaughterhouse, on three separate days. At evisceration, the entire reproductive tract was extracted and carefully inspected. The ovaries were examined and those tracts with a pre-ovulatory follicle ≥35 mm in diameter (as confirmed after opening the follicle using a scalpel blade), associated with uterine edema on examination of the opened endometrial surface (vivid endometrial folds with a gelatinous appearance), were sampled as pre-ovulatory (pre-OV). When the ovaries had evidence of a recent ovulation, as confirmed after sectioning the ovary to examine the presence of a corpus hemorrhagicum or juvenile corpus luteum (CL) identified as a luteal structure with a large, red central clot and a luteinized wall that was still visibly crenulated, the reproductive tracts were classified as post-ovulatory (post-OV) and also harvested. The oviduct and the attached ovary containing the pre-ovulatory follicle or the CL were separated from the uterus distally to the uterotubal junction, the ovary was dissected, and the oviduct was carefully dried with a tissue and placed into a Petri dish within ~30 min of slaughter. A non-heparinized hematocrit capillary tube (Merck, Madrid, Spain) attached to a 5-ml syringe by a silicone tube was inserted into the ampulla of the oviduct through the infundibular opening. Gentle aspiration was performed to recover fluid, and the fluid retrieved was expressed into 500-μl tubes. Aspiration was repeated for a total of at least three times per oviduct. The retrieved fluid was centrifuged for 2 min in a microcentrifuge at room temperature (RT) to remove large cellular masses. The supernatant was retrieved, transferred to a clean tube, and placed in dry ice until its arrival at the laboratory (4–5 h). Once at the laboratory, the OF was centrifuged at 16,000 × *g* at 4°C for 20 min and the supernatant transferred to a clean tube. The volume obtained was measured using a micropipette, and the samples were then kept at −80°C until analysis. A total of four pre-ovulatory OF samples and four post-ovulatory OF samples were submitted for proteomic analysis. Each sample was extracted from an individual female (*n* = 8 samples in total). Between 8 and 10 μl per sample was used for proteomic analysis.

### Protein Digestion and Tagging With the iTRAQ^®^ 8-Plex Reagent

After sample thawing, the total protein concentration of each sample was determined using the Pierce 660-nm protein assay kit (Pierce, Rockford, IL, USA). For digestion, 40 μg of protein from each replicate and condition (pre-ovulatory OF, *n* = 4; post-ovulatory OF, *n* = 4) was precipitated by the methanol/chloroform method. Protein pellets were resuspended and denatured in 20 μl of triethylammonium bicarbonate (TEAB; 7 M urea/2 M thiourea, 0.1 M, pH 7.5; SERVA Electrophoresis GmbH, Heidelberg, Germany), reduced with 1 μl of 50 mM Tris (2-carboxyethyl)phosphine (TCEP; AB SCIEX, Foster City, CA, USA), pH 8.0, at 37°C for 60 min, and followed by 2 μl of 200 mM cysteine-blocking reagent (methyl methanethiosulfonate, MMTS; Pierce) for 10 min at RT. The samples were diluted up to 120 μl to reduce the urea/thiourea concentration with 50 mM TEAB. Digestions were initiated by adding 2 μg of sequencing grade modified trypsin (Sigma-Aldrich, St. Louis, MO, USA) to each sample in a ratio of 1:20 (*w*/*w*), which were then incubated at 37°C overnight on a shaker. Sample digestions were evaporated to dryness.

Each trypsin-digested sample, previously reconstituted with 80 μl of 70% ethanol/50 mM TEAB, was labeled at RT for 2 h with a half unit of iTRAQ Reagent 8-plex kit (AB SCIEX, Foster City, CA, USA). Isobaric tags for relative and absolute quantification (iTRAQ) labeling was performed according to the following schema: iTRAQ 113 reagent: pre-ovulatory OF R1; iTRAQ 114 reagent: pre-ovulatory OF R2; iTRAQ 115 reagent: pre-ovulatory OF R3; iTRAQ 116 reagent: pre-ovulatory OF R4; iTRAQ 117: post-ovulatory OF R1; iTRAQ 118 reagent: post-ovulatory OF R2; iTRAQ 119 reagent: post-ovulatory OF R3; and iTRAQ 121 reagent: post-ovulatory OF R4. After labeling, the samples were combined and the reaction stopped by evaporation in a SpeedVac. Salts were washed using a Sep-Pak C18 cartridge (Waters, Milford, MA, USA).

### Liquid Chromatography and Mass Spectrometry Analysis

A 2-μg aliquot of each sample was subjected to 2D nano-liquid chromatography electrospray ionization tandem mass spectrometry (LC-ESI-MS/MS) analysis using a nano-liquid chromatography system (Eksigent Technologies NanoLC Ultra-1D Plus, AB SCIEX, Foster City, CA, USA) coupled to a high-speed TripleTOF 5600 mass spectrometer (SCIEX, Foster City, CA, USA) with a Nanospray III source. The injection volume was 5 μl. The analytical column used was a silica-based reversed-phase nanoACQUITY UPLC 75-μm ×15-cm column (Waters), with 1.7 μm particle size. The trap column was an Acclaim PepMap 100 column (ThermoFisher Scientific, Waltham, MA, USA), with 5 μm particle diameter and 100 Å pore size, switched on-line with the analytical column. The loading pump delivered a solution of 0.1% formic acid in water at 2 μl/min. The nano-pump provided a flow rate of 250 nl/min and was operated under gradient elution conditions using 0.1% formic acid in water as mobile phase A and 0.1% formic acid in acetonitrile as mobile phase B. Gradient elution was performed according to the following scheme: isocratic conditions of 96% A/4% B for 5 min, a linear increase to 40% B in 205 min, then a linear increase to 90% B for an additional 15 min, isocratic conditions of 90% B for 10 min, and a return to the initial conditions in 2 min. The total gradient length was 250 min.

Data acquisition was performed with the TripleTOF 5600 system. Ionization occurred under the following conditions: ion spray voltage floating (ISVF), 2,800 V; curtain gas (CUR), 20; interface heater temperature (IHT), 150; ion source gas 1 (GS1), 20; and declustering potential (DP), 85 V. All data were acquired using the information-dependent acquisition (IDA) mode with Analyst TF 1.7 software (AB SCIEX, Foster City, CA, USA). For the IDA parameters, a 0.25-s MS survey scan in the mass range of 350–1,250 Da was followed by 30 MS/MS scans of 150 ms in the mass range of 100–1,500 Da (total cycle time, 4.5 s). Switching criteria were set to ions greater than the mass-to-charge ratio (*m*/*z*) 350 and smaller than *m*/*z* 1,250, with a charge state of 2–5 and an abundance threshold of more than 90 counts per second. Former target ions were excluded for 20 s. The IDA rolling collision energy (CE) parameters script was used for automatically controlling the CE.

### Data Analysis and Statistics

The MS/MS spectra were exported to mgf format using Peak View v1.2.0.3 and searched using Mascot Server 2.5.1, OMSSA 2.1.9, X!TANDEM 2013.02.01.1, and Myrimatch 2.2.140 against a composite target/decoy database built from the *Equus caballus* reference proteome sequences at UniProt Knowledgebase (January 2020), together with commonly occurring contaminants. After recalibration of patent ion mass measurements using high-scoring X!TANDEM hits, the search engines were configured to match potential peptide candidates with mass error tolerance of 10 ppm and fragment ion tolerance of 0.02 Da, allowing for up to two missed tryptic cleavage sites and isotope error (^13^C) of 1, considering a fixed MMTS modification of cysteine and variable oxidation of methionine, pyroglutamic acid from glutamine or glutamic acid at the peptide N-terminus, acetylation of the protein N-terminus, and modifications of lysine, tyrosine, and peptide N-terminus with iTRAQ 8-plex reagents. Score distribution models were used to compute the peptide-spectrum match *p*-values (20), and spectra recovered by a false discovery rate (FDR) ≤ 0.01 (peptide-level) filter were selected for quantitative analysis. Approximately 5% of the signals with the lowest quality were removed prior to further analysis. Differential regulation was measured using linear models (21), and statistical significance was measured using *q*-values (FDR). All analyses were conducted using software from Proteobotics (Madrid, Spain).

## Results

Proteomic analysis resulted in the identification of a total of 1,173 proteins that were present in pre-ovulatory and post-ovulatory OF. Only the characterized proteins with at least one peptide and FDR <0.01 have been presented (Supplementary Table 1).

**Figure 1:**
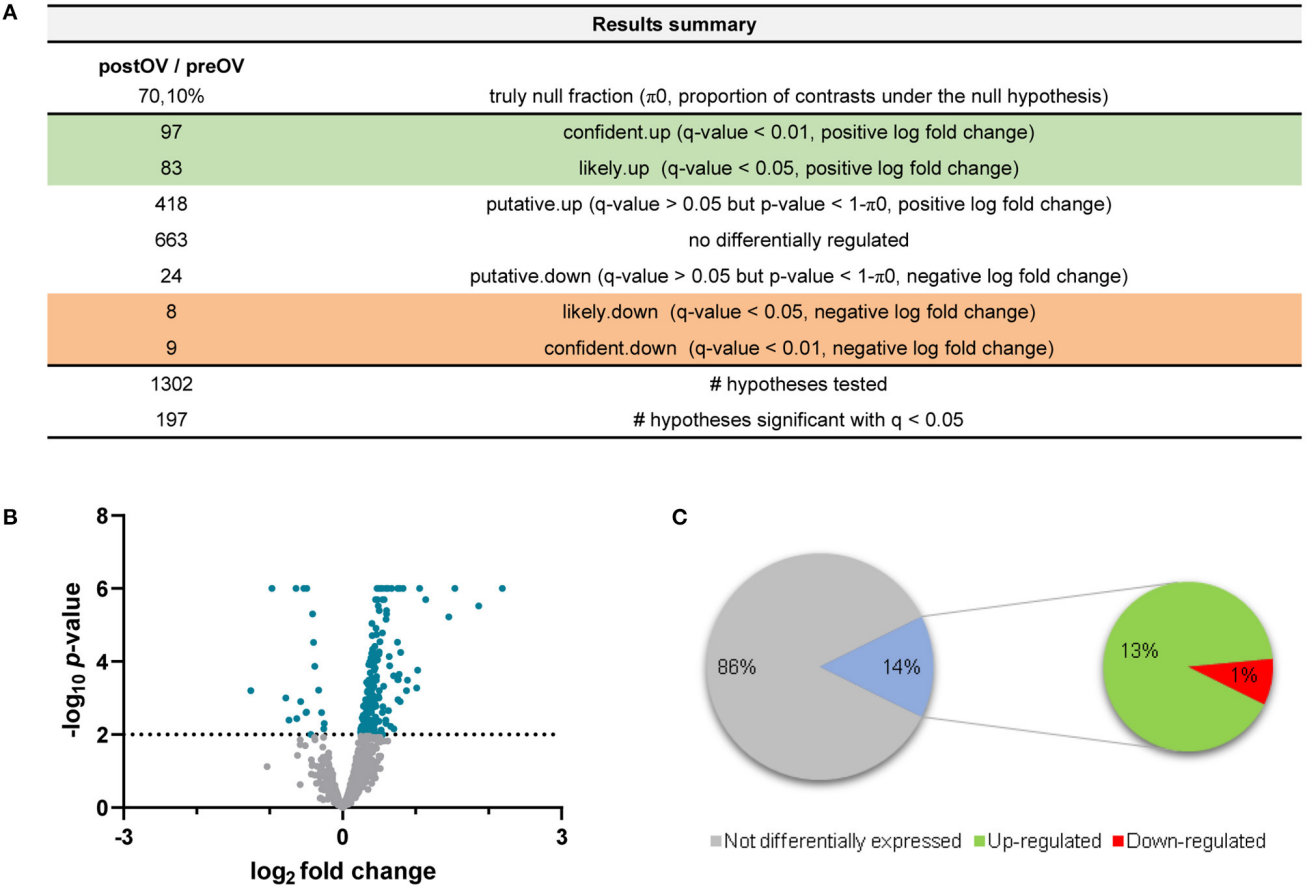
Summary of data analysis for the comparison of post-ovulatory oviductal fluid (post-OF) vs. pre-ovulatory oviductal fluid (pre-OF). **(A)** The number of expressed proteins (upregulated, downregulated, or non-differentially regulated—*left column*) and their degrees of confidence (*right column*) are represented. A total of 1,302 hypotheses were tested. Log2 fold changes and *q*-values (false discovery rate, FDR) were considered to identify the differentially expressed (upregulated and downregulated) proteins in the post-OF vs. the pre-OF group. **(B)** Volcano plot representing the differentially and non-differentially expressed proteins. Only proteins with *q* <0.05 and –log_10_ *p* > 2 were considered differentially expressed (*blue*). Positive log2 fold changes represent upregulated proteins and negative log2 fold changes represent downregulated proteins. **(C)** For ease of analysis in *Equus caballus* databases, repeated, unresolved, and uncharacterized proteins were removed. A total of 1,173 unique, inferred, and characterized proteins (listed in Supplementary Table 1) were finally analyzed according to their log2 fold changes and *q*-values (FDR). Proteins with *q*-values (FDR) > 0.05 were considered not differentially expressed (1,002 proteins, 86%). Proteins with *q* <0.05 were considered differentially expressed (171 proteins, 14%). Positive fold changes represent upregulated proteins (156 proteins, 13%), whereas negative fold changes represent downregulated proteins (15 proteins, 1%). The figures were created with Microsoft Excel and GraphPad Prism.

**Figure 2:**
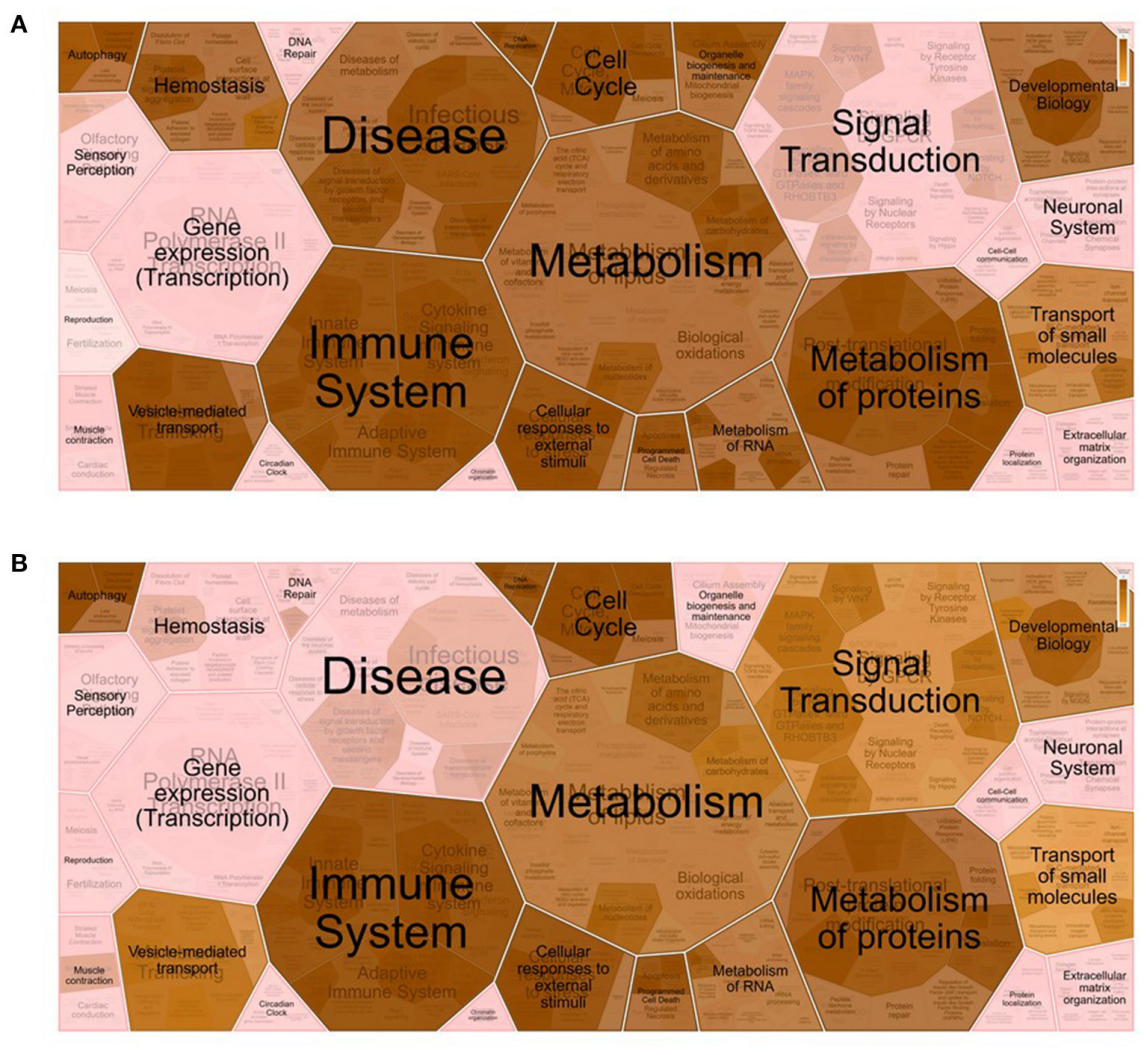
Voronoi pathway visualization (Reacfoam) for the identified proteins from equine oviductal fluid belonging to the Gene Ontology (GO) categories *extracellular exosome* (GO:0070062) **(A)** and *cytoplasm* (GO:0005737) **(B)**. A Reactome overrepresentation pathway analysis of the identified proteins listed in the above-mentioned GO categories (see Supplementary Table 2) was performed with the analysis tool of Reactome (https://reactome.org/) by exploring the corresponding gene names in the *Homo sapiens* database. The *p*-values are shown with the gradient from *pink* (*p* > 0.05) through *light brown* (0.05 < *p* <0) to *dark brown* (*p* ≈ 0).

For enrichment analyses, the UniProt^1^ accession numbers were associated with the gene names and Ensembl^2^ IDs by using the databases for *E. caballus* proteins, genes, and transcripts from DAVID Bioinformatics^3^ (22, 23) and g:Profiler^4^ (24). Enrichment analysis of the proteins identified in the OF was performed using the Functional Annotation Tool provided by DAVID Bioinformatics. Gene Ontology (GO) (molecular function, cellular component, and biological process), Kyoto Encyclopedia of Genes and Genomes (KEGG), and UniProt keywords were used as the annotation databases. Among the initial 1,173 proteins, the DAVID software recognized 691 unique proteins from the *E. caballus* species (Supplementary Table 2A). Enrichment analysis of these 691 proteins revealed that 54.3% (375 among the initial 691) were associated with the *extracellular exosome* category (GO:0070062) and 111 with the *cytoplasm* category (GO:0005737). Reactome^5^ pathway analysis of the identified proteins belonging to these two GO categories was performed by exploring the corresponding gene names in the *Homo sapiens* database (Supplementary Tables 2B,C). Additionally, Voronoi pathway visualizations (Reacfoam) (Figure 1) showed that proteins belonging to the categories *extracellular exosome* (GO:0070062) and *cytoplasm* (GO:0005737) were almost equally involved to pathways related to metabolism, immune system, cell cycle, and developmental biology, among others. On the other hand, proteins from the category *extracellular exosome* showed a unique involvement in the processes of hemostasis and organelle biogenesis and maintenance, while proteins from the category cytoplasm were involved in signal transduction processes. Moreover, proteins from *extracellular exosome* were associated with disease-related pathways. To represent protein changes in pre- and post-ovulatory equine OF, the iTRAQ results (1,173 characterized proteins) were analyzed to obtain log2 fold changes. Proteins from post-ovulatory OF were compared with the proteins from pre-ovulatory OF and were categorized as upregulated (positive log fold change) or downregulated (negative log fold change) (Figure 2). Only differentially expressed upregulated or downregulated proteins categorized as “likely” or “confident” (FDR <0.05 and FDR <0.01, respectively) and with *p* <0.05 were taken into consideration and are listed in Figure 1, Supplementary Table 3. Among the identified proteins, 15 were found to be downregulated in post-ovulatory OF compared to the pre-ovulatory counterpart and 156 were upregulated in post-ovulatory OF compared to pre-ovulatory OF.

**Figure 3:**
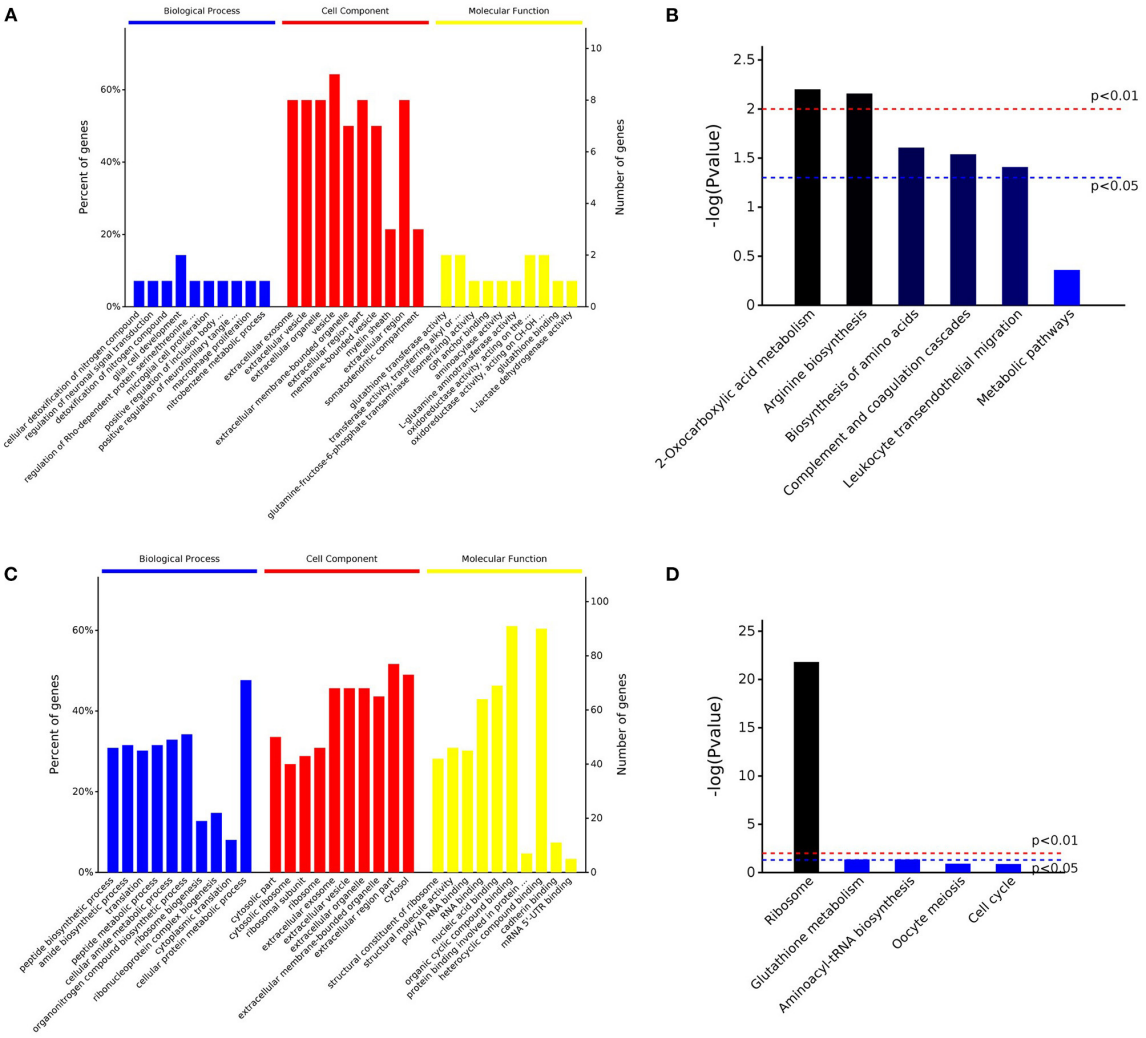
Overview of the enrichment analysis of the downregulated and upregulated proteins in post-ovulatory oviductal fluid (OF) vs. pre-ovulatory OF. Up to 10 significantly enriched terms in the biological process (BP), cellular component (CC), and molecular function (MF) categories of the Gene Ontology (GO) analysis for downregulated **(A)** and upregulated **(C)** proteins are shown. Terms of the same category are ordered by *p*-values (cutoff *p* = 0.05), the terms on the *left* being more significant. The *left* and *right y*-axes show the percentage and number, respectively, of the involved proteins in a term **(A,C)**. Five out of the top 10 enriched KEGG pathways for the downregulated **(B)** and upregulated **(D)** proteins are shown. The significance of the results, based on genome background enrichment, is shown by *broken red* (*p* = 0.01) and *blue* (*p* = 0.05) *lines* **(B,D)**.

Enrichment analysis of the upregulated and downregulated proteins was performed on the corresponding Ensembl IDs using the g:GOSt Functional profiling tool^6^ of g:Profiler. Ensembl IDs were examined as ordered query in the *E. caballus* database, taking into consideration only annotated genes. GO molecular function, cellular component, and biological process and KEGG were used as the annotation databases (Supplementary Tables 4A, 5A). Furthermore, pathway analyses of the downregulated and upregulated proteins in equine post-ovulatory vs. pre-ovulatory OFs were performed exploring the corresponding gene names in the *H. sapiens* database (Supplementary Tables 4B, 5B). Representative pictures of the enriched GO and KEGG categories in downregulated and upregulated proteins were obtained uploading gene symbols in the *E. caballus* database of the bioinformatics tool Omicsbean^7^ (Figure 3).

Interestingly, when the enrichment analysis was performed for the 15 downregulated proteins (Supplementary Table 4A), seven of them (47% of the proteins) were included in the category *metabolic pathways* (KEGG:01100). The downregulated proteins under this category were aminoacylase 1 (ACY1), glutathione S-transferase Mu 1 and 3 (GSTM1 and GSTM3, respectively), glutamine–fructose-6-phosphate aminotransferase 1 (GFPT1), NME/NM23 nucleoside diphosphate kinase 1 (NME2), lactate dehydrogenase B (LDHB), and phosphoglycerate dehydrogenase (PHGDH).

**Figure 4:**
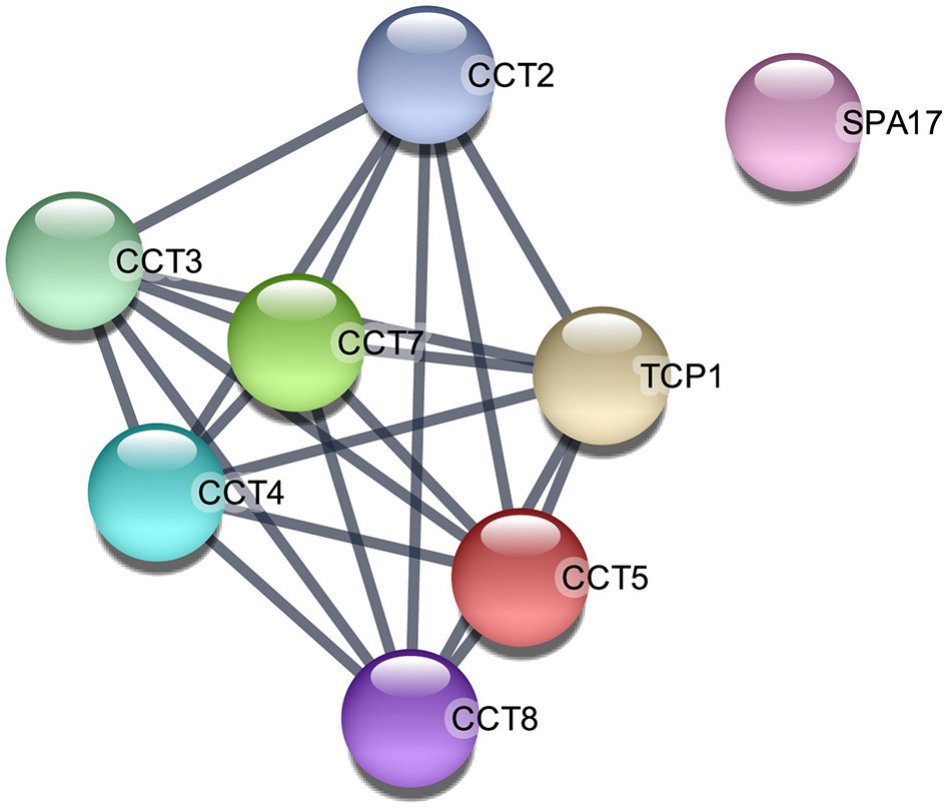
Protein–protein interaction network among the upregulated proteins in post-ovulatory oviductal fluid belonging to the category single fertilization (GO:0007338). *Lines between nodes* indicate edges (representing protein–protein associations) with the highest confidence (0.900). The image was created with the STRING app for Cytoscape. *CCT2*/*CCT3*/*CCT5*/*CCT7*/*CCT8*, chaperonin-containing TCP1 subunits 2, 3, 5, 7, and 8, respectively; *SPA17*, sperm autoantigenic protein 17; *TCP1*, T-complex 1.

On the other hand, when the 154 upregulated proteins were investigated using an enrichment analysis approach, the main categories identified were directly related to metabolism (Supplementary Table 5A). In the *metabolic process* category (GO:0008152), 116 proteins (75% of the initial 167 upregulated transcripts) were enclosed, *organic substance metabolic process* (GO:0071704) involved 110 proteins (71%), and *cellular metabolic process* (GO:0071704) included 109 proteins (71%), while in *nitrogen compound metabolic process* (GO:0006807) and *primary metabolic process* (GO:0044238), 107, and 105 proteins were included, respectively. Of note is that 35% of the proteins were involved in *peptide metabolic process* (GO:0006518), suggesting an active protein synthesis and remodeling in the post-ovulatory phase. Moreover, 124 (81%) post-ovulatory upregulated proteins were localized in the *cytoplasm* (GO:0005737), in particular in the *cytosolic ribosome* (GO:0005840; 31%). Interestingly, eight upregulated proteins in post-ovulatory OF were grouped into the categories *single fertilization* (GO:0007338), *sperm–egg recognition* (GO:0035036), and *binding of sperm to zona pellucida* (GO:0007339). The proteins involved in these categories were the T-complex 1 (TCP1) and its chaperonin-containing TCP1 subunits 2, 3, 5, 7, and 8 (CCT2, CCT3, CCT5, CCT7, and CCT8, respectively), together with sperm autoantigenic protein 17 (SPA17). Moreover, five out of these eight proteins were under the *category zona pellucida receptor complex* (GO:0002199). STRING^8^ was used to investigate the protein–protein interaction networks. Protein names were searched in the *H. sapiens* database as multiple protein query. STRING revealed that all these proteins, except SPA17, were also specifically associated with the highest edge confidence (Figure 4), so it may be assumed that they contribute to a shared function. In addition, the Reactome pathway analysis of the upregulated proteins showed their involvement in the categories *metabolism of proteins* (R-HSA-392499), *cellular responses to stress* (R-HSA-2262752), and *developmental biology* (R-HSA-1266738), among others (Supplementary Table 5B). An enrichment analysis of the upregulated proteins belonging to these categories was performed with GO, confirming their involvement not only as *structural constituent of ribosome* (GO:0003735) and in *peptide metabolic process* (GO:0006518) (Supplementary Table 5C) but also in *reactive oxygen species metabolic process* (GO:0072593) (Supplementary Table 5D).

It is worth mentioning that among the upregulated proteins, there were albumin (ALB), the secretoglobin SCGB1A1, versican (VCAN), myosin heavy chain 9 (MYH9), and gelsolin (GSN).

## Discussion

The present work explores the proteome of equine OF in the peri-ovulatory period. Our data reveal that consistent differences exist in the composition of equine OF obtained from oviducts ipsilateral to a pre-ovulatory follicle and the one obtained after ovulation, coinciding with previous results in bovine (25). Interestingly, the enrichment analysis revealed that, among the 691 unique proteins recognized for *E. caballus*, 375 (54.3%) were associated with the *extracellular exosome* category (GO:0070062). Hence, the majority of the identified proteins in equine OF may be involved in cell-to-cell communication as the identified protein cargo may be delivered from the oviductal milieu to the gametes/embryos in membrane-enclosed microparticles, as also demonstrated in other species (18, 26). The second category obtained in the enrichment analysis was *cytoplasm* (GO:0005737; 200 identified proteins), which corresponds with 28.9% of all the identified proteins, as also described in other species in which 13–27% of all the identified proteins were shown to have a cytoplasmic origin (18). The cytoplasmic origin of the proteins could be related to epithelial cell breakdown during OF extraction, or, most likely, they could be derived from apocrine or non-canonical secretory pathways (27, 28). Interestingly, these cytoplasmic proteins have also been described to have an exosomal origin (28). In our samples, the enrichment analysis revealed that 111 proteins identified as having a cytoplasmic origin (55.5%) were also enclosed in the *extracellular exosome* category (data not shown). Hence, it is likely that this cytoplasmic protein cargo is delivered by extracellular vesicles to the gametes and embryos.

The Reactome pathway analysis showed that, in the proteins identified as upregulated in post-ovulatory OF, 87 of them were included in the *metabolism of proteins* pathway and 56 were enclosed in the *developmental biology* pathway, revealing an intense protein turnover during early embryo development in horses, as demonstrated in other species (29). Interestingly, regarding the 15 downregulated proteins found in post-ovulatory OF, eight of them exhibited an FDR <0.01 and were classified as confident downregulated, namely, FABP3, CLU, ACY1, GFPT1, LDHB, GSTM3, NME2, and TAGLN2 (Supplementary Table 3).

The fatty acid-binding protein 3 FABP is part of a protein family known as fatty acid-binding proteins, in which nine isoforms are enclosed. These proteins bind to long-chain fatty acids (C16–C20) and transport them to intracellular compartments (into the peroxisome, mitochondria, or the endoplasmic reticulum) or to the extracellular milieu, free, or enclosed in extracellular vesicles (30).

Isoform 3 of FABP is called FABP3 or heart FABP and has been demonstrated to be involved in aberrant lipid accumulation in bovine oocytes subjected to *in vitro* maturation (31). Interestingly, Smits et al. (32) showed that equine embryos produced *in vitro* showed lower messenger RNA (mRNA) expression of *FABP3* compared to *in vivo*-derived embryos, indicating that lipids could be a potential energy source for the embryo during the pre-implantation window. The lower expression of FABP3 in post-ovulatory OF compared to pre-ovulatory OF may also be linked to the fact that FABP3 overexpression induces apoptosis, as demonstrated in heart and embryonic cancer cells, and thus, its expression needs to be finely regulated when the embryo enters the oviduct (33). In porcine oviductal cells cultured *in vitro*, the changing mRNA expression of *FABP3* over the culture time has also been described, highlighting that the oviduct has the ability to modulate fatty acid metabolism. This modulation by oviductal cells could avoid possible lipotoxic effects favoring early embryo quality and survival (34), which can also explain our findings.

Regarding GFPT1, this enzyme controls the flux of glucose into the hexosamine pathway, being determinant for the production of hyaluronic acid. GFPT1 is significantly overexpressed in *in vivo* matured equine cumulus cells compared to the *in vitro* matured counterparts, explaining in part the vivid cumulus expansion observed in equine oocytes produced *in vivo* (35). In the porcine oviduct, it has been demonstrated that hyaluronic acid modulates sperm capacitation and enhances sperm survival, delaying the capacitation process (36). Hence, the lower expression of GFPT1 in equine post-ovulatory OF could be related to a physiological change in the oviductal milieu in which capacitation needs to be induced to achieve oocyte fertilization. In this sense, lactate dehydrogenase B (LADHB) is involved in lactate synthesis from pyruvate, and it is known that lactate is a core metabolite found in equine OF (11). Lactate is required to maximize mitochondrial activity and motility in equine spermatozoa incubated under capacitating conditions (37) and is also required during *in vitro* oocyte maturation (38, 39). Hence, the downregulation of LADHB in post-ovulatory OF compared to pre-ovulatory OF could also be related to the specific lactate needs of equine gametes during capacitation and fertilization. Similarly, clusterin (CLU) may also be playing a role modulating sperm capacitation in the oviduct. CLU is a chaperone found in the extracellular space and in various body fluids secretions, including equine OF (19, 40). In rabbits, CLU expression increases in the mixture of spermatozoa and OF retrieved from the oviduct 4 h post-insemination during the pre-ovulatory period (41). CLU has also been demonstrated to undergo re-localization in mice during capacitation (42) and could be supporting the final maturation process of spermatozoa in the equine oviduct, as previously demonstrated in rabbits (40).

Another protein present in the OF is GSTM3, an antioxidant enzyme involved in cell protection against oxidative stress that has been found in the uterine fluid of pregnant and non-pregnant mares (43). This protein has been detected in goat sperm surface and plays a crucial role as a zona pellucida-binding protein (44); hence, as our data and previous reports in sheep (45) show a GSTM3 downregulation in post-ovulatory OF compared to pre-ovulatory OF, this protein could be playing a crucial role in gamete interaction promoting fertilization.

The protein TAGLN2 belongs to the transgelin (TAGLN) family, which comprised three isoforms and have been identified as actin-binding proteins, which are known to stabilize the actin cytoskeleton (46). TAGLN2 is known to affect actin cross-linking blocking F-actin depolymerization and has been described to play a core role in embryo implantation in mice (46). However, equine embryos undergo fixation around day 21 post-ovulation, so the downregulation of TAGLN2 expression in post-ovulatory OF could be instead modulating actin depolymerization during sperm capacitation, promoting acrosome reaction, as previously reported in other species (47), or may be playing another role that needs to be further explored.

Nucleoside diphosphate kinase B (NME2) plays a major role in the synthesis of nucleoside triphosphates other than adenosine triphosphate. It has also been identified as a potential canonical transcription factor that regulates gene transcription through its DNA-binding activity (48) and has been described to be overexpressed in bovine OF ipsilateral to the pre-ovulatory follicle (25) in the form of extracellular vesicle cargo (49). However, the role that nucleoside diphosphate kinase B may play in equine fertilization remains to be studied. This is the same scenario for ACY1, a soluble homodimeric zinc-binding enzyme that is involved in the hydrolysis of *N*-acetylated proteins. The *N*-acylation of a protein usually leads to the extension of its half-life, and interestingly, 50–80% of all cellular proteins show formylated or acetylated N-termini. After the degradation of proteins, free amino acids can be recycled by the enzymatic hydrolysis of *N*-acylated amino acids catalyzed by aminoacylases, such as ACY1 that has a wide substrate specificity (50). Amino acid metabolism is crucial for embryo development, and a different amino acid turnover has been demonstrated to happen in the culture medium of human embryos resulting in clinical pregnancy and those that underwent reabsorption (51). Once more, the exact role that this protein may play in equine OF remains to be further studied.

In our setting, the most significantly upregulated protein was secretoglobin (SCGB1A1), coinciding with previous studies in horses in which this uteroglobin was significantly upregulated in the ipsilateral oviduct of pregnant mares (19). A similar secretoglobin (SCGB1D2) was already found to be upregulated in the human oviduct in the early luteal phase, and both secretoglobins have been associated with anti-inflammatory/immunomodulatory, anti-chemotactic, and embryonic growth-stimulatory activities, as also proposed in horses (19, 52). The upregulated VCAN was demonstrated to promote cell motility and migration (53, 54), suggesting that the post-ovulatory oviductal microenvironment is involved in the migration process of the zygote toward the uterus. ALB upregulation in post-ovulatory OF has already been described in equine (19), while GSN was found in bovine oviductal exosomes playing a core role in sperm–oviduct interaction and early embryo development (49). Moreover, the upregulation of MYH9 in post-ovulatory OF is in agreement with a previous study on bovine post-ovulatory OF in which this protein was demonstrated to be a specific sperm-interacting protein (55). Regarding the upregulated proteins in the category *single fertilization* (GO:0007338), it has already been reported that the molecules involved in the process of sperm–egg recognition and binding are not only expressed in the spermatozoa or oocytes but can also be dispersed in OF (56). According to the STRING analysis, TCP1 and its subunits CCT2, CCT3, CCT5, CCT7, and CCT8 interact in common pathways (Figure 4). This result is in accordance with previous studies that hypothesized that chaperone proteins are necessary to deliver and assemble multiprotein complexes on the surface of gametes before sperm–egg interaction (57).

Our data reveal interesting differences in the proteome of equine OF prior to and post-ovulation. These findings may help to continue unraveling which factors promote fertilization and early embryo development, aiming to improve *in vitro* fertilization outcome in horses. More studies are required to achieve the proposed goal, and further studies will be carried out to keep improving our understanding regarding the physiology of oocyte fertilization in horses.

## Data Availability Statement

The datasets presented in this study can be found in online repositories. The names of the repository/repositories and accession number(s) can be found at: http://www.proteomexchange.org/, PXD025320.

## Ethics Statement

Ethical review and approval was not required for the animal study because samples were retrieved from a slaughterhouse.

## Author Contributions

PF-H contributed to the investigation, methodology, writing the original draft, manuscript review, and editing. FM helped with the data curation, formal analysis, methodology, writing the original draft, and in manuscript review and editing. MS-C contributed to the investigation and in manuscript review and editing. LJG-M and MJB helped with manuscript review and editing and in funding acquisition. LG-F did the formal analysis, investigation, methodology, writing the original draft, and in manuscript review and editing. BM-G contributed to the conceptualization, data curation, formal analysis, funding acquisition, investigation, methodology, project administration, supervision, writing the original draft, and in manuscript review and editing. All authors contributed to the article and approved the submitted version.

## Conflict of Interest

The authors declare that the research was conducted in the absence of any commercial or financial relationships that could be construed as a potential conflict of interest.

## Publisher's Note

All claims expressed in this article are solely those of the authors and do not necessarily represent those of their affiliated organizations, or those of the publisher, the editors and the reviewers. Any product that may be evaluated in this article, or claim that may be made by its manufacturer, is not guaranteed or endorsed by the publisher.

## Footnotes

^1^ https://www.uniprot.org/

^2^ https://www.ensembl.org/index.html

^3^ https://david.ncifcrf.gov/

^4^ https://biit.cs.ut.ee/gprofiler/convert

^5^ https://reactome.org/

^6^ https://biit.cs.ut.ee/gprofiler/gost

^7^ http://www.omicsbean.cn/

^8^ https://string-db.org/
